# Single nucleotide polymorphisms in interleukin-6 attenuates hepatocytes injury in hypoxia/re-oxygenation via STAT3 signal pathway mediated autophagy

**DOI:** 10.1007/s11033-020-06090-2

**Published:** 2021-01-23

**Authors:** Hongwei Tang, Hongbo Fang, Wenzhi Guo, Shengli Cao, Danfeng Guo, Huapeng Zhang, Jie Gao, Shuijun Zhang

**Affiliations:** 1 Department of Hepatobiliary and Pancreatic Surgery, The First Affiliated Hospital of Zhengzhou University, Zhengzhou, China; 2 Open and Key laboratory of Hepatobiliary & Pancreatic Surgery and Digestive Organ Transplantation at Henan Universities, Zhengzhou, Henan People’s Republic of China; 3 ZhengZhou Key Laboratory of Hepatobiliary & Pancreatic Diseases and Organ Transplantation, Zhengzhou, Henan People’s Republic of China

**Keywords:** IL-6, Ischemia/reperfusion injury, Autophagy, STAT3 signaling pathway

## Abstract

Ischemia-reperfusion injury (IRI) is inevitable during liver surgery, and it is an important factor affecting the prognosis of patients. *IL-6* rs1800796 single nucleotide polymorphisms (SNPs) can promote synthesis and secretion of IL-6 and protect hepatocytes from IRI. In this study, we investigated the mechanisms by which IL-6 alleviates hepatic IRI. We transfected lentivirus which carries *IL-6* rs1800796 to L02 cells and constructed the cell line (L02-IL6) with a high expression of *IL-6*. The biological function of IL-6 SNPs was explored through a cell model of hypoxia-reoxygenation (H/R). Cell viability was evaluated by CCK8 and Real-Time Cell Analysis (RTCA), and found that the viability of the L02-IL6 cells was higher than that of the control group (*P* < 0.01). Flow cytometry assay showed that the rate of apoptosis was significantly decreased in L02-IL6 cells. Furthermore, in comparison with the control group, the level of cleaved-caspase3, which is an important marker of apoptosis, was dramatically decreased. These differences showed that the sequence variants at rs1800796 of the IL-6 gene could improve the resistance against H/R. Moreover, the levels of autophagy-related proteins, such as LC3 and Beclin-1, were upregulated in L02-IL6 group on H/R injury, which means IL-6 could alleviate apoptosis via activating the autophagy pathway. And we also found that the STAT3 signal pathway was activated. Next, we investigated whether the exogenous treatment with IL-6 affect hepatocytes and thus play a protective role. We pre-treated the L02 cells with recombinant human IL-6 for 12 h and then made H/R treatment. We found the treatment with 100 ng/ml IL-6 alleviated the damage of L02 cells and inhibited the apoptosis. And the further study revealed the pre-treatment with IL-6 activated the STAT3 signaling pathway in the L02 cells and then caused the activation of autophagy and apoptosis inhibition. IL-6 might play a critical role in alleviating hepatic IRI, through its modulation of the STAT3 signaling pathway, and activation of autophagy. Recombinant human IL-6 might be a potential therapeutic target in hepatic IRI.

IRI is an inevitable process during liver surgery, including liver resection and transplantation. And, it is also the most important prognostic factor for patients after liver resection, especially in liver transplantation [1–3]. Many studies with IRI have shown that it might lead to graft failure in about 10% of patients after transplantation as well as increase the risks of graft rejection and liver dysfunction [4]. The potential factors and mediators involved in IRI are numerous, so elucidating the mechanism of IRI and effective pharmacological treatments that could alleviate IRI would have important clinical significance.

Interleukin-6 (IL-6) is a multifunctional cytokine, that plays a major role in regulating the inflammatory or immunomodulatory response [5]. And it has been found that IL-6 can protect the liver against IRI and promote the proliferation of hepatocytes [6–10]. The liver is the primary source of IL-6 and meanwhile the main area for the clearance of IL-6 [11]. IL-6 has been widely believed to be conducive to the induction of acute-phase response, promoting the synthesis and secretion of acute phase proteins (APPs), accelerating hepatocyte proliferation. [12]. For murine models of liver injury, the injection of the IL-6 could attenuate the lesion of the liver. In contrast, the murine models without the treatment with the IL-6 were vulnerable to the IRI. In fatty liver rat models of IRI, treatment with IL-6 for 10 days before the induced liver injury could ease the hepatic steatosis and IRI. [13]. IL-6 is known to derive from the secretion of the immunocytes that infiltrates into the liver injury area and the innate immune cells of the liver. It protects the liver mainly via the mediation against some inflammatory factors such as tumor necrosis factor α (TNF-α) and so on [14–16]. Mounting evidence has shown that the high expression of IL-6 in the liver graft is related to the prognosis of the liver transplantation patients, and the IL-6 rs1800796 in the graft has an effect on the metabolism of Tacrolimus, an immunosuppressive drug [17]. Likewise, the construction of the cell line with a high expression level of IL-6 rs1800796 can ameliorate the IRI in L02 cells [18]. Generally, IL-6 seemed directly concerned with the production of anti-apoptotic signals by inducing B cell lymphoma-2 (Bcl-2) related protein family members [19]. Therefore, we assume that the sequence variant of rs1800796 SNPs may induce hepatocytes to secrete IL-6 in an autocrine way following the IRI and the elevated IL-6 can directly affect the hepatic cells and protect them against the IRI. Thus, to verify the hypothesis, we transfected L02 cells with lentivirus, that could secrete more IL-6. The effect of IL-6 or its SNPs were explored by a cell model of H/R.

## Materials and methods

### Materials

L02 cells were purchased from Procell Life Science &Technology (Cat No.CL-0111). Recombinant human IL-6 (rh-IL6) was produced by PeproTech (Cat No.200–06). Lentivirus-NC and Lentivirus-IL6 were got from GenePharma. Caspase3 (9662), cleaved-caspase3 (9664), LC3A/B (4108), Beclin-1 (3495), Stat3(4904), Phospho-stat3 (9145), GAPDH(5174) antibodies were got from Cell Signaling Technology. Annexin V-FITC/PI (556547) was obtained from BD Biosciences. Fetal bovine serum (FBS) was got from Gibco. Dulbecco’s Modified Eagle Medium (DMEM,12100–500) was got from Solarbio. E-plates were purchased from ACEA Biosciences. Cell Counting Kit-8 (CCK8, CK04) was got from Dojindo Laboratories. ALT (C009–2) was got from Nanjing Jiancheng Bioengineering Institute. Myco-blue ® Mycoplasma Detection (D101) was purchased from Vazyme Biotech. And the following machines used: tri-gas incubator (NBS Galaxy 48R, USA), normoxic incubator (Thermo Scientific), Real-Time Cell Analysis (RTCA, ACEA Biosciences Inc.), Flow cytometer (BD Biosciences, USA), Varioskan Lux (Thermo Scientific).

### Cell culture and H/R model

L02 cells were cultured in DMEM with 10% FBS and 1% penicillin-streptomycin at 37 °C with 5% CO_2_ in a humidified incubator_._ The results of mycoplasma detection showed that culture cells were not contaminated by mycoplasma. For H/R, the culture medium was replaced with glucose and serum-free medium and placed in a tri-gas incubator with 1% O_2_,5% CO_2_ and N_2_ for indicated periods. Following hypoxia, the culture medium was replaced with the normal medium and placed in a normoxic incubator.

### Stable cell transfection

After the formation of a confluent epithelial monolayer, the cells were digested with trypsin and then plated in a 24-well culture plate at a density of approximately 2 × 10^4^cells/well for 24 h. Then we replaced the original culture medium with fresh medium with 5 μg/ml polybrene and added an appropriate dose of virus suspension to culture the cells for 24 h. Following it, we changed the new culture medium to continue to culture the cells for 48 h. We then observed the cells with a fluorescence microscope and screened stable cell lines with puromycin.

### Real-time cell analysis (RTCA)

After the formation of a confluent epithelial monolayer, the cells were digested with trypsin and then plated in a 16-well E-Plate with 50 μl culture medium. Next, the E-Plate was placed on the RTCA Station and the Background was checked to ensure all the wells in a normal situation, and the Cell Index was lower than 0.063. Then the E-Plate was taken out and added 100 μl homogenized cell suspension, and then was placed at room temperature for 30 min. Next, the E-Plate was again put on the RTAC Station in the incubator for real-time dynamic detection of cell proliferation. After 24 h, the E-Plate 16 was taken out, the original medium was replaced to the sugar-free and serum-free medium and the cells subjected to hypoxia were monitored in real time in a tri-gas incubator (1% O2). Subsequently, the sugar-free and serum-free medium was replaced with fresh medium in the E-Plate 16, and the state of the cells under the reoxygenation condition was continuously monitored.

### Determination of hepatocyte injury

Hepatocyte injury was evaluated by measuring the concentration of alanine aminotransferase (ALT) in the cell supernatant, CCK8, and apoptosis detection using apoptosis assays kit. A total of 5 × 10^3^ cells were seeded into the 96-well culture plates for 24 h. Subsequently, the culture medium was replaced with glucose and serum-free medium and subjected to H/R. The level of ALT in the supernatants was tested according to the manufacturer’s instructions. And then the cells were incubated with CCK-8(10ul/well) for 1–4 h. The absorbance was determined at a wavelength of 450 nm. Apoptotic cells were evaluated by the flow cytometry (FCM) through Annexin V-FITC/PI staining according to the instruction.

### Western blot analysis

Cells were lysed with RIPA on ice for 20 min. After centrifuging, the total protein was quantified by Bicinchoninic acid (BCA). And then, the protein was separated on SDS–PAGE gels. After transferring onto NC membranes., the membranes were blocked with defatted milk for 3 h and then incubated with different primary antibodies at 4 °C overnight. After washing with TBST 3 times, the membranes were incubated with the Secondary Antibodies for 1 h. proteins were visualized via Licor Odyssey® CLx Imaging System.

### Statistical analysis

All data was statistically analyzed by using the statistical analysis software SPSS 19.0. For comparing values, the student^,^s t-test was used. A *p* value of <0.05 was considered statistically significant.

## Results

### Construction of L02-IL6 cell lines

**Fig. 1:**
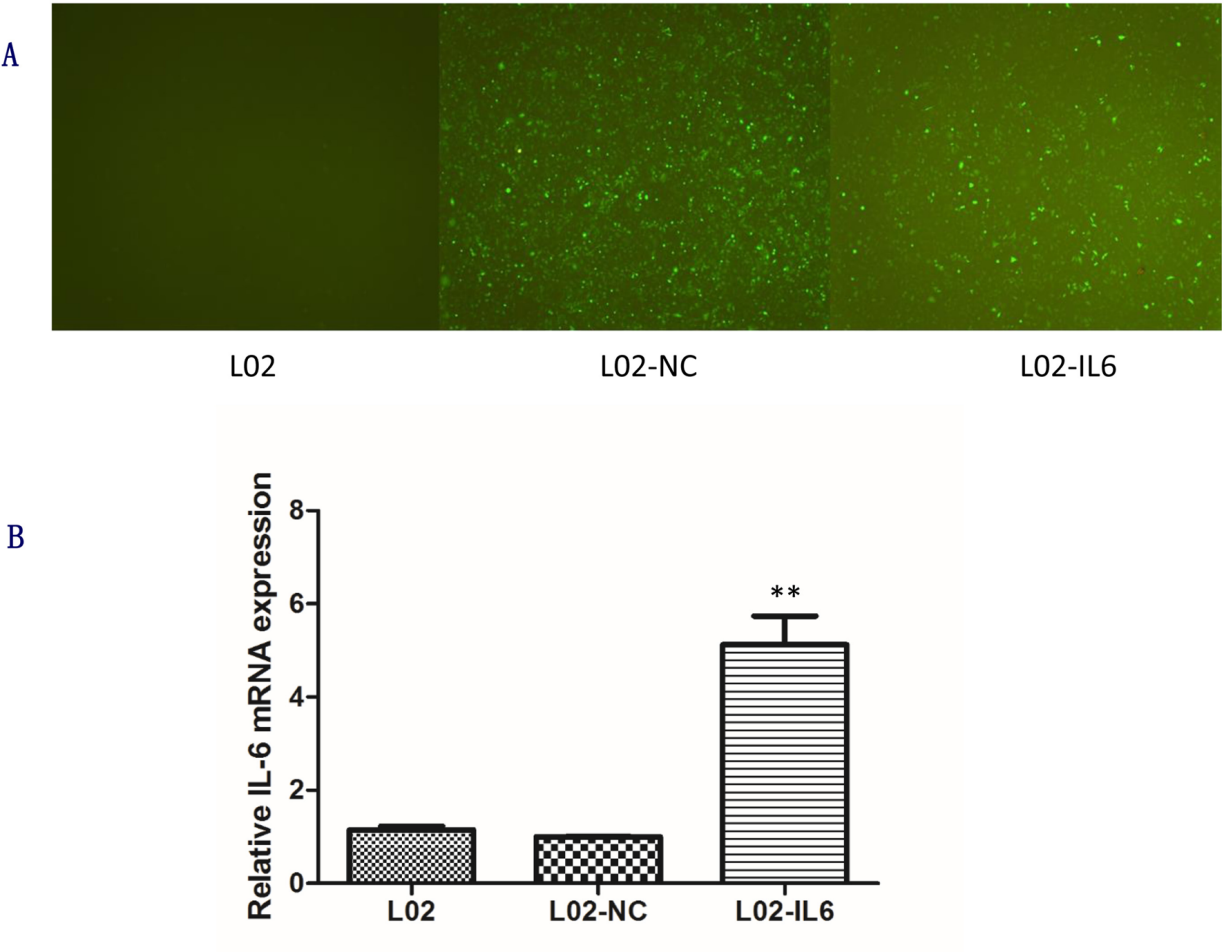
Images of L02 cells and relative expression of *IL-6*. (**a**) Fluorescent image of L02 cells at 48 h after lentiviral transduction. (**b**) Relative expression of IL-6 examined by RT-qPCR after transfection of the LO2 cells with lentiviral particles of LV5-NC and LV5-*IL6*. All data are representative results of three independent experiments. **P < 0.01 compared with L02-NC group. IL-6, interleukin-6; L02-NC, LO2 cells transfection with LV5-NC

In order to assess the effect of the variants of the *IL-6* SNPs during hepatocyte H/R injury, we successfully constructed the L02-IL6 cells that stably transfected with IL-6 overexpression lentivirus particles. Meanwhile, the L02-NC cells, which served as a negative control, were transfected with LV5-NC particles according to the manufacturer’s instructions. The cells transfected with lentivirus emitted green fluorescence, and the transfer efficiency of lentiviral vector was approximately 70% (Fig. 1a). Following 0.8μg/ml puromycin selection, stable highly expressing IL-6 cell lines were constructed. Additionally, the relative expression of IL-6 in L02-IL6 cells was much more than that in L02-NC and normal L02 cells (*p* < 0.01) (Fig. 1b).

### Effect of IL-6 on H/R injury in L02 cells

**Fig. 2:**
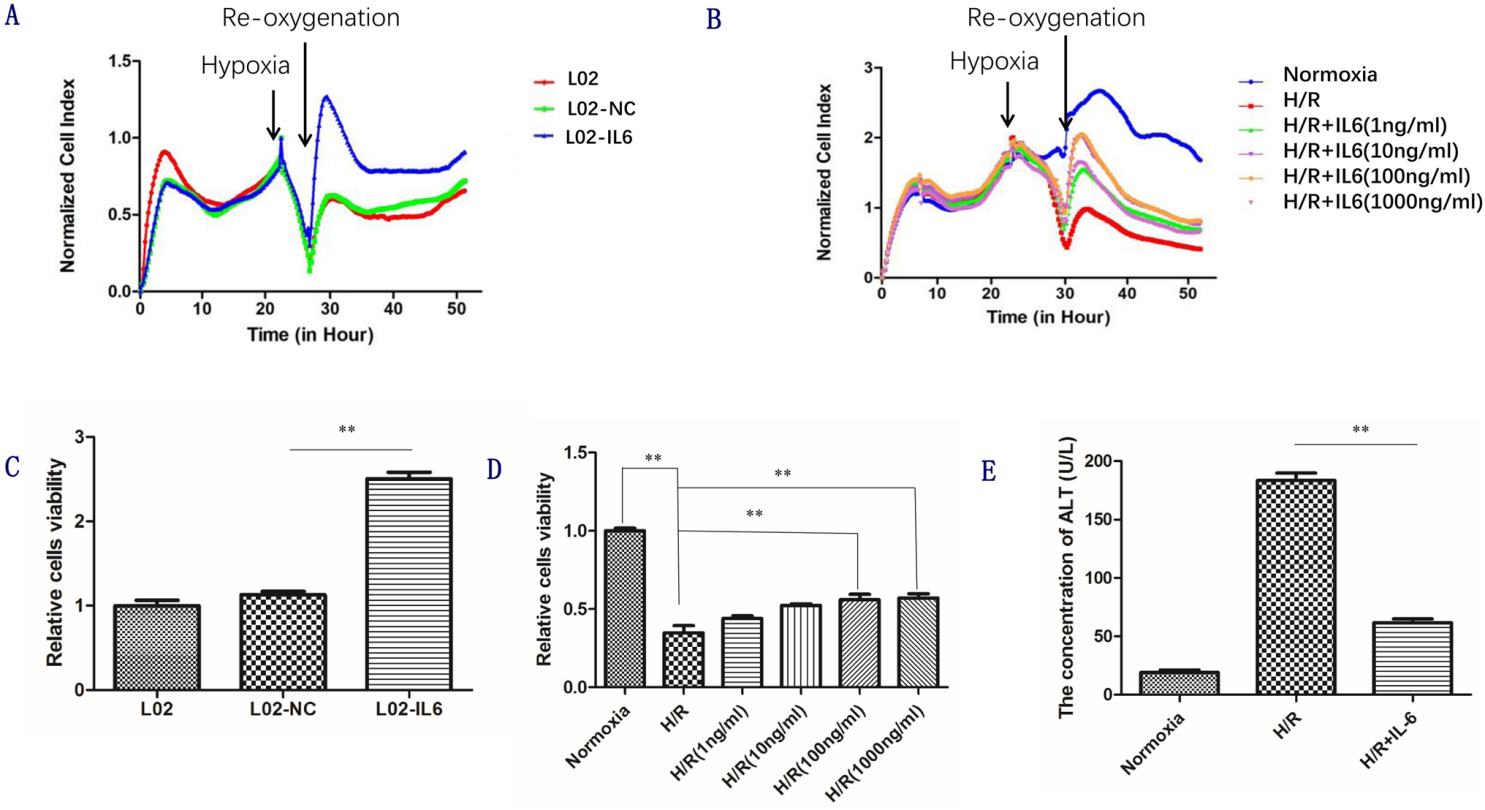
IL-6 dramatically alleviate H/R injury. (**a**, **b**) IL-6-action curve got from RTCA analysis during H/R injury (**c**, **d**) Cell viability was determined by CCK8 assay. (**e**) Concentrations of AlT in the supernatant of L02 cells in normoxic, H/R group and H/R + IL6 groups. All data are representative results of three independent experiments.**P < 0.01. Normoxia, the cells cultured under normal oxygen; H/R, hypoxia/reoxygenation; H/R + IL-6, pretreatment with IL-6 at a dose of 100 ng/ml for 12 h, and then subjected to H/R; ALT, alanine aminotransferase

We used RTCA to assess cell activity. Following H/R injury, the viability of L02-IL6 cells was much more increased compared to the L02-NC and normal L02 cells. There was no significant difference between L02-NC and normal L02 cells, which means that the titer of lentiviral had no cytotoxicity. Additionally, we found that the greatest difference in the cell viability occurred at 4 h of reperfusion (Fig. 2a). These differences were mainly caused by the sequence variants at rs1800796 of the *IL-6* gene. We also analyzed whether IL-6 treatment alleviated H/R injury in L02 cells. By RTCA analysis, the results indicated that compared to the untreated control, L02 cells pretreated with IL-6 for 12 h could tolerate the hypoxia treatment for a longer time and could return to its natural state immediately in the context of re-oxygenation. L02 pretreated with IL-6 at a dose of 100 ng/ml, showed better effect at 4 h of reperfusion when compared to others (Fig. 2b). Next, we chose the time point of 4 h after reperfusion as the time index and further verified the result mentioned above with the CCK8 experiment. The L02 viability was in a dose-dependent manner. The cell viability increased as the concentration of the IL-6 increased. There were no obvious differences in cell viability between H/R-IL6 (100 ng/ml) and H/R-IL6 (1000 ng/ml) (Fig. 2c, d). In addition, the concentration of ALT in the H/R-IL6 (100 ng/ml) group was much lower than that in the H/R group (Fig. 2e).

### IL-6 alleviates apoptosis via potentiating STAT3 Activation on H/R injury

**Fig. 3:**
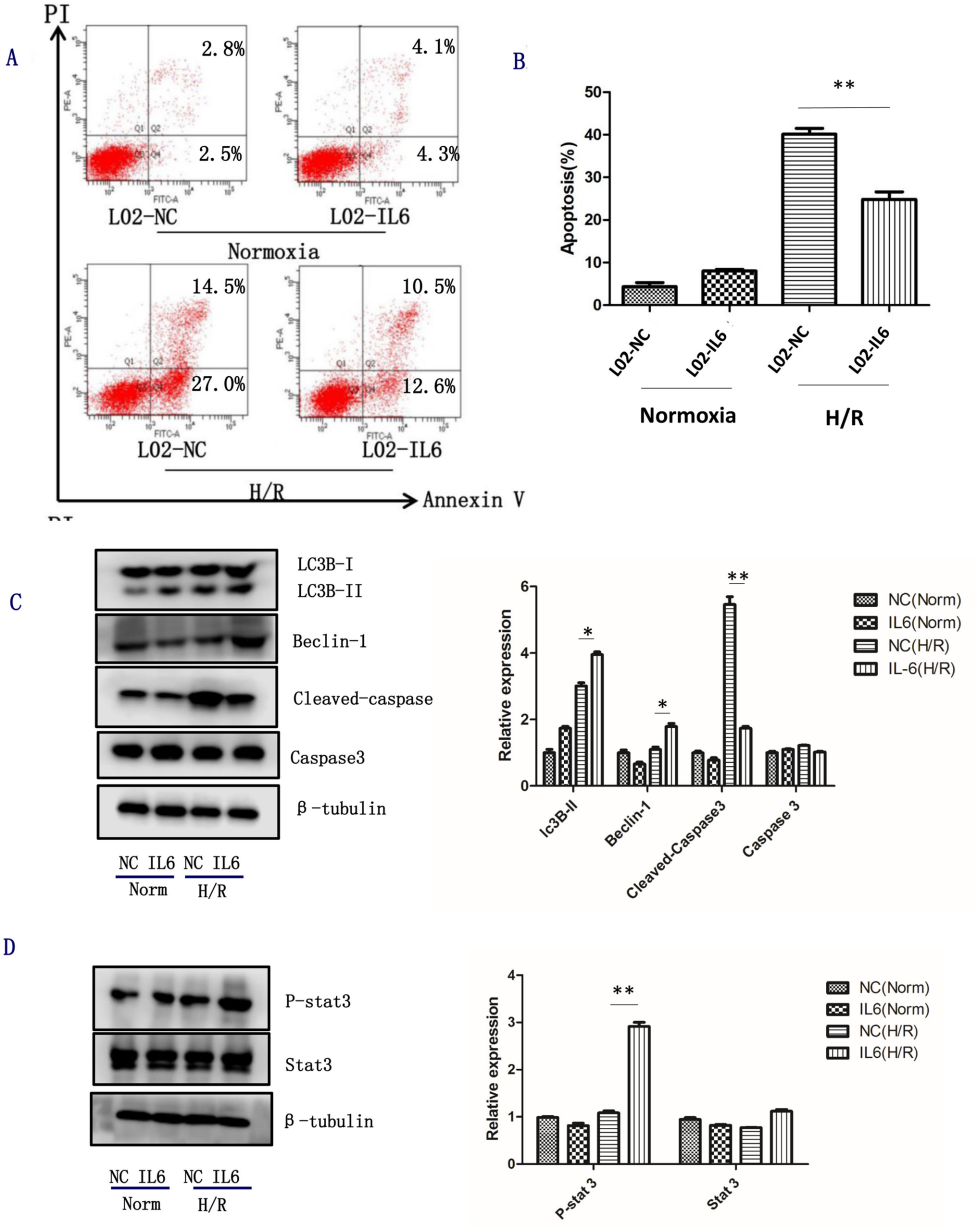
The variants of the IL-6 SNPs could alleviate apoptosis via potentiating STAT3 activation on H/R injury. L02 cells were transfected with LV5-NC and LV5-*IL6,* (**a**) Apoptosis was determent by Annexin-V-FITC/PI staining and flow cytometry assay. (**b**) A representative figure was shown for each treatment. (**c**) The expression of Caspase 3, Cleaved-caspase3, Beclin-1, LC3 were examined by western blotting analysis. (**d**) The level of Stat3 and p-stat3 were determined by western blotting analysis. β-Tubulin was used a loading control. All data are representative results of three independent experiments. NC, L02-NC; IL6, L02-IL6; Norm, Normoxia; H/R, hypoxia/reoxygenation

Apoptosis is the key element of IRI. We here investigated whether IL-6 could alleviate apoptosis on H/R injury. We treated L02-IL6 cells and L02-NC cells with H/R and then stained with Annexin V-FITC/PI. We found that the hypoxia-reoxygenation could increase the percentage of Annexin V-FITC-positive cell subpopulation of L02-IL6 cells and L02-NC cells. And the rate of apoptosis in L02-IL6 cells was significantly decreased than that in the L02-NC cells (Fig. 3a, b). Western blotting analysis showed that the variants of the *IL-6* SNPs could decrease the expression of Cleaved-caspase 3, which is an important marker of apoptosis. To investigate whether the autophagy pathway was involved in the anti-apoptosis of L02-IL6 cells, we detected the protein that associated with autophagy, and found that the expression of LC3 and Beclin-1 was markedly increased in L02-IL6 group on H/R injury (Fig. 3c). These results indicated that IL-6 could alleviate apoptosis via activating the autophagy pathway. IL-6 could directly induce STAT3 phosphorylation in hepatocytes. In this study, we found that the level of phosphor-STAT3 was much higher in the L02-IL6 group than that in the L02-NC group (Fig. 3d).

**Fig. 4:**
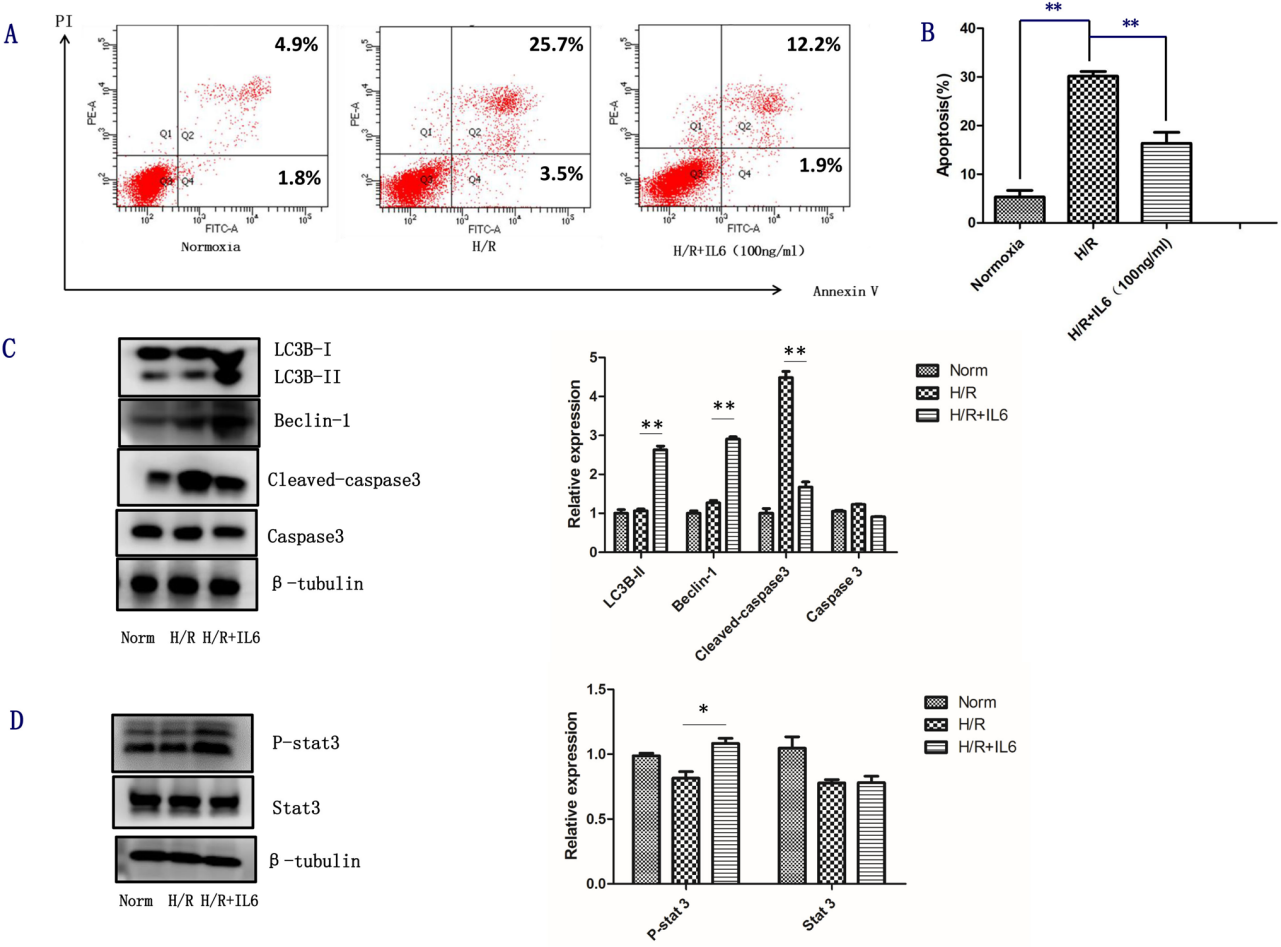
Exogenous IL-6 alleviate apoptosis via potentiating STAT3 Activation on H/R injury. L02 cells were pretreated with IL-6 at a dose of 100 ng/ml for 12 h. (**a**) Apoptosis was determent by Annexin-V-FITC/PI staining and flow cytometry assay. (**b**) A representative figure was shown for each treatment. (**c**) The expression of Caspase 3, Cleaved-caspase3, Beclin-1, LC3 were examined by western blotting analysis. (**d**) The level of Stat3 and p-stat3 were determined by western blotting analysis. β-Tubulin was used a loading control. All data are representative results of three independent experiments. Norm, Normoxia; H/R, hypoxia/reoxygenation; H/R + IL-6, pretreatment with IL-6 at a dose of 100 ng/ml for 12 h

Moreover, following H/R, we pretreated L02 cells with rh-IL6 at a dose of 100 ng/ml for 12 h. We found that the use of IL-6 could dramatically decrease the percentage of Annexin V-FITC (+) cell subpopulation by 15.1% (Fig. 4a, b). And rh-IL6 could inhibit the activation of Caspase3, and the autophagy pathway was significantly activated after H/R (Fig. 4c). Besides, rh-IL6 could also induce STAT3 phosphorylation (Fig. 4d).

## Discussion

Ischemia/reperfusion injury plays a major role in affecting the prognosis of patients with liver resection, transplantation, liver trauma, and hemorrhagic shock [1, 20]. Interleukin 6 (IL-6) is a member of chemokine secreted mainly by macrophages, Th2 cells, and endothelial cells, which exerts more than one function [21]. IL-6 is essential for maintaining hepatic tissue homeostasis, and it is also a potent hepatocyte protective agent [22]. Although substantial progress has been made, there are still many important problems to be solved, in particular those concerning the effect of IL-6 of liver under the physiological and pathological conditions that deserve our attention. Moreover, the variants of the *IL-6* SNPs were investigated in healthy people [23]. Moreover, it has been reported that the variants of the *IL-6* on rs1800796 could affect the metabolism rate of tacrolimus by improving liver regeneration after liver transplantation [17]. Allele C of *IL-6* rs1800796 could promote the secretion of IL-6 [18, 24]. Therefore, we constructed L02-IL6 cells, which means *L-6* rs1800796 locus SNPs (G → C), that express more IL-6 and the negative control group, L02-NC. Since the hepatocytes hardly secrete IL-6, we never silenced the expression of IL-6. Subsequently, the cells were subjected to H/R. We found that the variants of *IL-6* SNPs could enhance the ability of hepatocytes to resist H/R injury. We also found that pretreated L02 cells respectively with various concentrations of IL-6 could also significantly alleviate H/R injury.

There are three forms of cell death in liver ischemia-reperfusion injury: necrosis, apoptosis, and autophagy. It is considered that apoptosis is a common pathological basis for several liver diseases, and the hepatocyte apoptosis is regulated by the activation of autophagy [25]. Autophagy is a natural process as the cell aging or cellular stress occurs, during which the damaged protein and organelle surrounded by the autocrine vesicles with bilateral membrane structure are transported to the lysosome for degradation to achieve the cyclic utilization. In the ischemia/reperfusion injury caused by the liver transplantation, the ischemic process is characterized by the autophagy activation, and reperfusion injury displays the autophagy inhibition [26]. Under this condition, the hepatocytes’ identification capacity for self-protein and organelle decreases, which causes a decrease of the autophagy and liver regeneration. However, autophagy activation can accelerate the recovery of liver injury caused by physical damage, alcohol, and food [27]. We treated L02 with H/R and found that the treatment with H/R inhibited the autophagy activity of L02 cells. It was consistent with the research result of Khader et al. [26]. Lee SC et al. [28] found that the immunosuppressor, Everolimus, could up-regulate the expression of autophagy markers (LC3B and P62) and reduced the expression of the pro-apoptotic proteins (cleaved-caspase 3 and cleaved PARP). When the autophagy was blocked, the anti-apoptotic effect of Everolimus vanished. So it could be concluded that Everolimus is a kind of potential drug used to treat IRI in liver transplantation, which can alleviate IRI by activating autophagy. It follows that autophagy plays a major role in liver IRI and the timely activation of autophagy of the cells can reduce liver injury. Therefore, we speculated that IL-6 may protect the hepatic cells against IRI by activating the autophagy of hepatocyte. To verify the hypothesis, we performed *IL6* rs1800796 locus SNPs (GC) mutations in L02-IL6ells and found that it increased the expression of autophagy marks (LC3A/B and Beclin-1) in L02 cells, and decreased the rate of apoptosis. Exogenous administration of IL-6 (long-time treatment) can achieve the same effect. These data suggest that IL-6 can protect hepatocytes by activating autophagy in hepatocytes when the ischemia-reperfusion injury occurs.

In this study, we also investigated the underlying mechanism of how IL-6 induced autophagy. STAT3, known as the downstream signal molecules of IL-6, had been investigated in a variety of models of liver injury [29]. Given the crucial role of STAT3 in hepatoprotective, it has been widely recognized the hepatocyte injury may result from the inhibition of STAT3 activation. And this hypothesis has been supported by the studies demonstrating that the lack of STAT3 activation is correlated with impaired liver regeneration [8, 30]. Treatment with IL-6 reversed these abnormalities and normalized regeneration. In this study, we found that IL-6 could trigger phosphorylation of stat3. These findings suggested that activation of the antiapoptotic STAT3 signal may be an important mechanism in the hepatoprotective effect of IL-6 in IRI.

In summary, our results show that under the H/R condition, sequence variants of *IL-6* rs1800796 locus SNPs (G → C) may protect hepatocytes from damages of hypoxia/reoxygenation. Moreover, both the paracrine and autocrine IL-6 can directly affect the hepatocytes and activate the anti-apoptotic STAT3 signal to protect the liver from the H/R injury. Above all, IL-6 could be a potential therapeutic for patients involving IRI.
